# Supplementary material for: High‐Throughput MicroED for Probing Ion Channel Dynamics
Source: Adv Sci (Weinh). 2025 May 29;12(30):e04881. doi: 10.1002/advs.202504881 (PMC12376597; doi:10.1002/advs.202504881)
Supplement: Supplementary file 1 — Supporting Information [file ADVS-12-e04881-s001.docx]

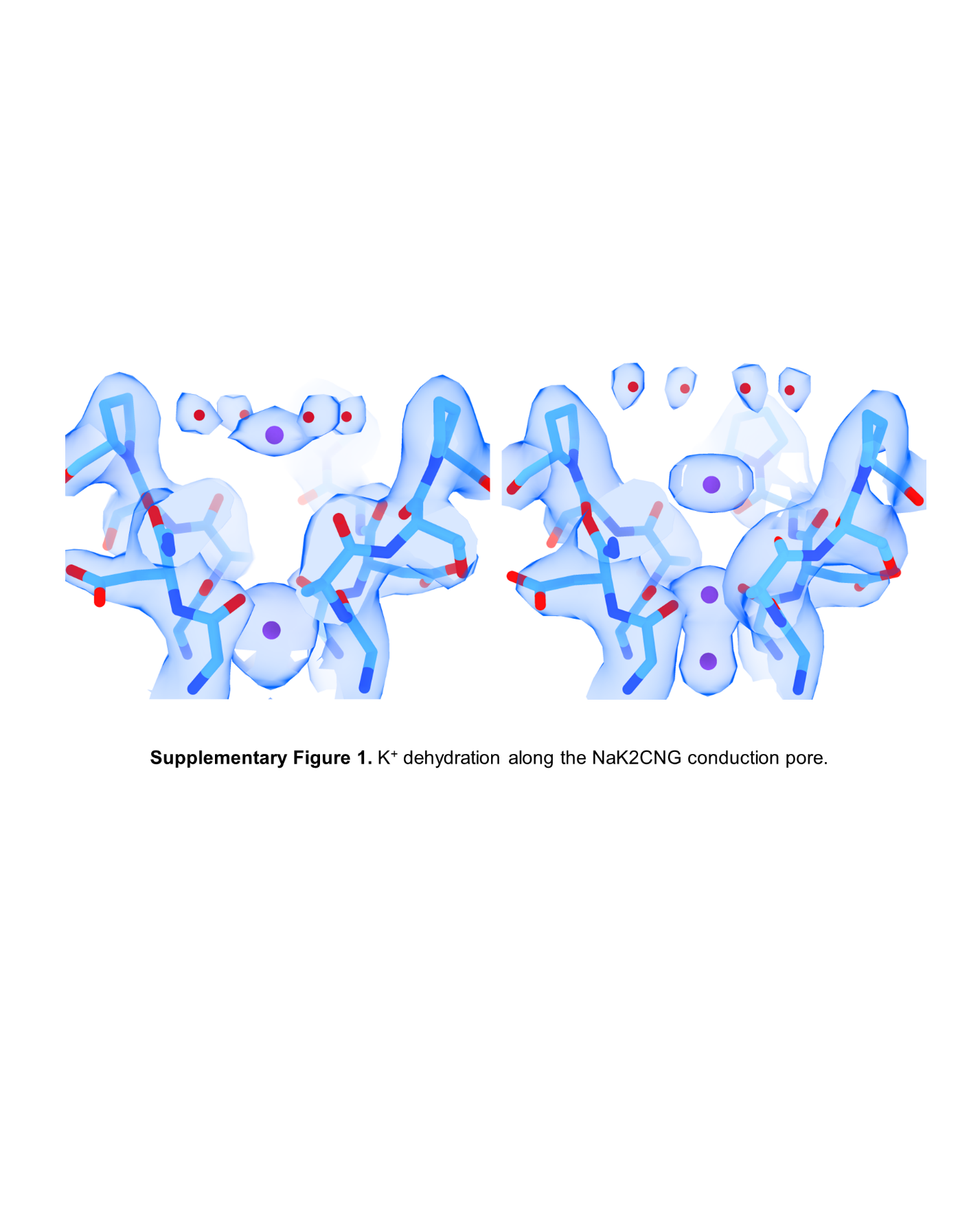


**Supplementary Figure 1.** K^+^ dehydration along the NaK2CNG conduction pore.


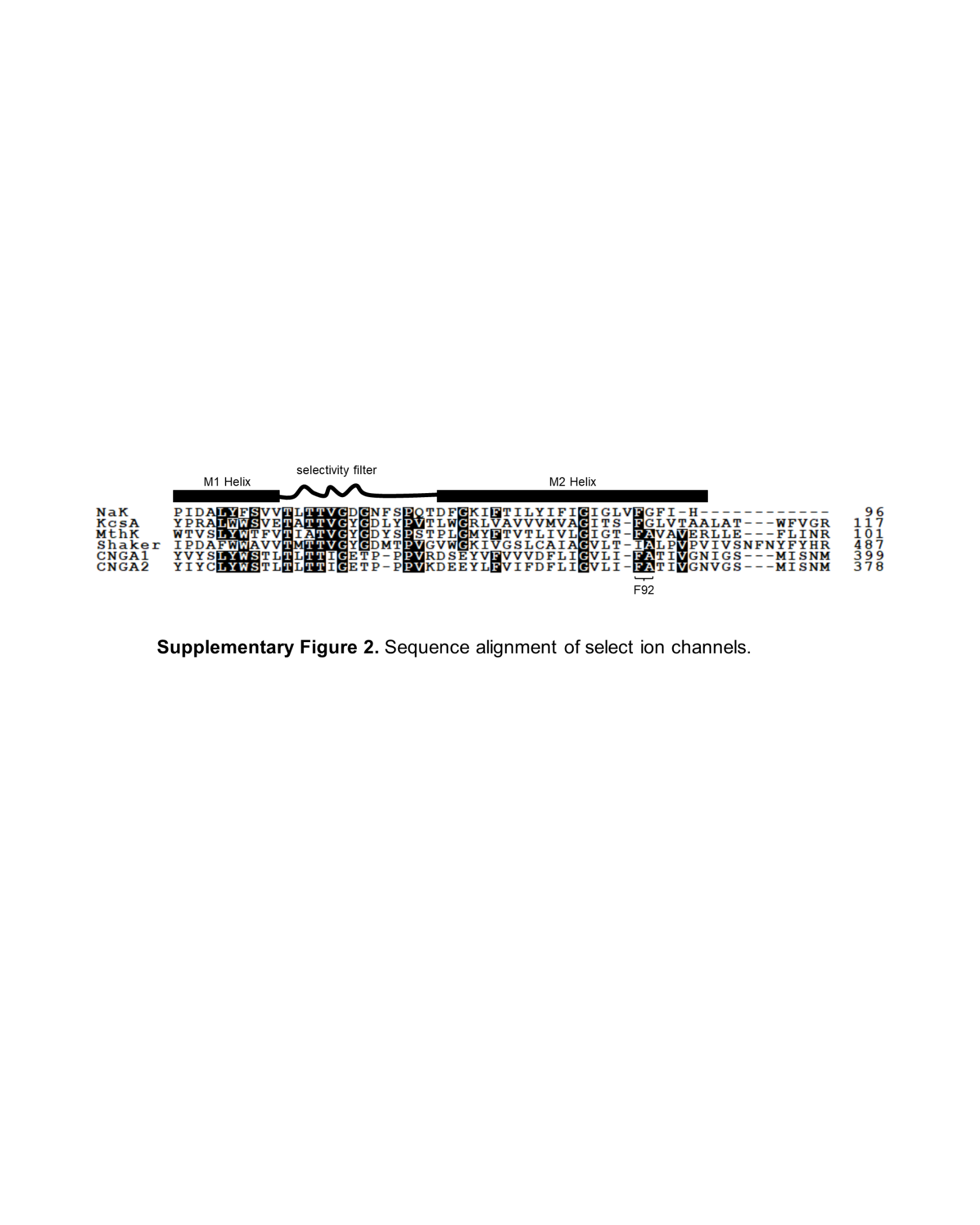


**Supplementary Figure 2.** Sequence alignment of select ion channels.
